# Identification of Secondary Metabolites by Multi-Omics Methods

**DOI:** 10.3390/metabo14110597

**Published:** 2024-11-06

**Authors:** Xin Fang

**Affiliations:** Key Laboratory of Phytochemistry and Natural Medicines, Kunming Institute of Botany, Chinese Academy of Sciences, Kunming 650201, China; xinfang@mail.kib.ac.cn

Plant natural products, also known as plant specialized metabolites (SMs) due to their lineage-specific distribution, are small molecules synthesized by plants to adapt to changing environments. These compounds are widely used as drugs, food supplements, dyes, and so on [1]. As such, isolation of individual metabolites from plant material and the application of appropriate biological assays is still a common method to evaluate their function. However, the low content of these compounds in plants promoted a synthetic biology approach to synthesizing them in living organisms. These technologies provided solid evidence for the elucidation of the structure of individual natural molecules and revealing their function, but they failed to identify a complex metabolite profile in a living organism that reflects a snapshot of changes. Recently, the emergence of omics, such as transcriptomics and metabolomics, can systematically scan changes in metabolites in vivo, providing an unbiased view of biological processes, thus facilitating the rapid elucidation of the in vivo function of these molecules. This Special Issue, “Identification of Secondary Metabolites by Multi-Omics Methods”, contains five original research articles from different countries, including traditional isolation and function evaluation of SMs (contributions 1 and 5), biosynthesis of valuable SM by engineered tobacco (contribution 3), and metabolomics-based omics analysis of SMs in plants under different conditions (contributions 2 and 4).

The identification of a complex chemical structure of a plant secondary metabolite is the first step toward the elucidation of its function. Although a large number of natural products have been extracted from plants, in recent decades, there have been serious problems with the separation of natural products from plants, such as high rates of repeated discovery and low rates of finding new compounds. At present, mass spectrometry-based metabolomics is the most commonly used method for studying secondary metabolites in plants, and the mass spectrometry data of metabolomics can be obtained through database analysis. Mass spectrometry molecular networks have emerged as a powerful tool that can rapidly eliminate known compounds from highly mixed metabolomes, homologous compounds, and other metabolites [2]. Alternatively, research has mainly focused on finding novel natural products produced by plants from rare species or in special habitats, such as hot springs and volcanic craters. Accordingly, Zhu et al. (contribution 1) isolated and characterized a new lathyrane type diterpenoid, as well as eleven known compounds from Euphorbia stracheyi Boiss, which is one of a few Euphorbia plants located in high-altitude alpine meadow regions. Among the identified known compounds, ionones are considered high-altitude adapted products as they are degraded from carotenoids that are key constituents in helping plants adapt to high-altitude-associated high UV light and low temperatures. 

Once enough amounts of phytochemicals are obtained, the bioactive assay will be performed to evaluate their active effects, usually by blind screening methods. For medicinal plants with traditional usage backgrounds, rational active screening and activity tracking programs based on their backgrounds could be a more effective approach. Guided by this idea, Yu et al. (contribution 5) isolated Dodonaea viscosa saponin B (DVSB) from the seeds of Dodonaea viscosa, whose crude showed good antifeedant activity on pests. They further found that DVSB has good combined activity with azadirachtin, one of the best natural pesticides, against one of the most pervasive pests, Spodoptera litura. 

Notably, most specialized metabolites are accumulated in low content in wild plants, such as taxol, yielding only 0.035 of the extracted dry weight of a yew tree [3]. To meet pharmaceutical demand, a large quantity of wild plants was overexploited, leading to depletion in the population of the species in natural habitats. Metabolic engineering is a promising approach to ensuring a reliable and sustainable supply of these valuable natural products. Therefore, Ren et al. (contribution 3) used cytoplasmic engineering strategies and the enhancement of isoprenoid precursors to produce miltiradiene, the key intermediate of tanshinones in engineered Nicotiana benthamiana. This study proposes an alternative platform for microbial systems that can be used for synthetic biology research on high-value plant specialized metabolites. 

Metabolites are closely related to the phenotype of organisms and serve as a bridge linking genotype and phenotype. Therefore, metabolomics research has a wide range of applications in life sciences, including phenotype analysis, gene function identification, and response to biotic or abiotic stress. Compared to microorganisms and mammals, plant metabolomics is particularly important because the compounds in plants are more diversified. For example, nearly 5000 metabolites have been detected in Arabidopsis, which is twice the number of metabolites in mammals and nearly 1500 more than that in microorganisms [4]. The reported stresses mainly include temperature stress, sulfur stress, nitrogen stress, phosphorus stress, oxidative stress, and multiple stresses [5]. Many different types of metabolites are involved in the stress response, including sugars such as sorbitol and fructan, fatty acid metabolites, and various amino acid metabolites. Some small-molecule substances, such as ascorbic acid, glutathione, anthocyanins, carotenoids, and other metabolites, are reposted to resist oxidative stress. In these regards, Cadena-Zamudio et al. (contribution 2) and Pan et al. (contribution 4) reported nitrate deprivation and altitudinal variation in the metabolic responses of Arabidopsis thaliana and Asarum sieboldii, respectively. It was shown that under N-starvation conditions, plants prefer survival over growth by analyzing the metabolomes from the nia1/nia2 double mutant and WT Arabidopsis plant. Meanwhile, in high-altitude areas, the Asarum roots mainly activate defense mechanisms by accumulating sugars, amino acids, and organic acids, while sugars in the leaves are reduced and nitrogen metabolism is enhanced. These results provide a theoretical basis for explaining the physiological and metabolic response mechanisms of plants to N-starvation and altitude changes.

In conclusion, the research papers in the Special Issue “Identification of Secondary Metabolites by Multi-Omics Methods” provide valuable foundations for plant secondary metabolites finding, activity evaluation, biosynthesis by synthetic biology approach, and in planta function. Although multi-omics methods have been widely used in plant secondary metabolite’s function [6,7,8,9,10,11,12,13], traditional purification, isolation, and structure elucidation are still needed to identify less explored unknown plant small molecules and provide a more solid basis for metabolomics investigation.

## Data Availability

Not applicable.
